# Muscle pain sensitivity after glutamate injection is not modified by systemic administration of monosodium glutamate

**DOI:** 10.1186/s10194-015-0546-0

**Published:** 2015-07-22

**Authors:** Akiko Shimada, Eduardo Castrillon, Lene Baad-Hansen, Bijar Ghafouri, Björn Gerdle, Malin Ernberg, Brian Cairns, Peter Svensson

**Affiliations:** Section of Orofacial Pain and Jaw Function, Department of Dentistry, Faculty of Health Sciences, Aarhus University, Aarhus, Denmark, Vennelyst Boulevard 9, 8000 Aarhus C, Denmark; Rehabilitation Medicine, Department of Medical and Health Sciences, Faculty of Health Sciences, Linköping University, & Pain and Rehabilitation Centre, County Council of Östergötland, SE 581 85 Linköping, Sweden; Occupational and Environmental Medicine, Department of Clinical and Experimental Medicine, Faculty of Health Sciences, Linköping University, and Centre of Occupational and Environmental Medicine, County Council of Östergötland, SE 581 85 Linköping, Sweden; Section of Orofacial Pain and Jaw Function, Department of Dental Medicine, Karolinska Institutet, 17177 Huddinge, Sweden; Faculty of Pharmaceutical Sciences, The University of British Columbia, 2405 Wesbrook Mall, Vancouver, British Columbia V6T 1Z3 Canada; Scandinavian Center for Orofacial Neurosciences (SCON), Vennelyst Boulevard 9, 8000 Aarhus C, Denmark

**Keywords:** Monosodium Glutamate, Myofascial temporomandibular disorders, Muscle pain sensitivity

## Abstract

**Background:**

Monosodium glutamate (MSG) is often thought to be associated with headache and craniofacial pains like temporomandibular disorders. This randomized, double-blinded, placebo-controlled study was performed to investigate how ingestion of MSG affects muscle pain sensitivity before and after experimentally induced muscle pain.

**Methods:**

Sixteen healthy adult subjects participated in 2 sessions with at least 1-week interval between sessions. In each session, two injections of glutamate (Glu, 0.5 M, 0.2 ml) and two injections of saline (0.9 %, 0.2 ml) into the masseter and temporalis muscles, respectively, were undertaken, with a 15 min interval between each injection. Injections of saline were made contralateral to Glu injections and done in a randomized order. Participants drank 400 mL of soda mixed with either MSG (150 mg/kg) or NaCl (24 mg/kg, placebo) 30 min before the intramuscular injections. Pressure pain thresholds (PPT), autonomic parameters and pain intensity were assessed prior to (baseline) and 30 min after ingestion of soda, as well as 5 min and 10 min after the intramuscular injections and at the end of the session. Whole saliva samples were collected prior to and 30, 45, 60, and 75 min after the ingestion of soda.

**Results:**

MSG administration resulted in a significantly higher Glu level in saliva than administration of NaCl and was associated with a significant increase in systolic blood pressure. Injections of Glu were significantly more painful than injections of NaCl. However, ingestion of MSG did not change the intensity of Glu-evoked pain. Glu injections also significantly increased systolic and diastolic blood pressure, but without an additional effect of MSG ingestion. Glu injections into the masseter muscle significantly reduced the PPT. However, pre-injection MSG ingestion did not significantly alter this effect. Interestingly, PPT was significantly increased in the trapezius after MSG ingestion and intramuscular injection of Glu in the jaw muscles.

**Conclusion:**

The main finding in this study was that systemic intake of a substantial amount of MSG does not influence either pain intensity or pressure pain sensitivity in the masseter and temporalis muscles into which Glu injections were made.

## Background

We have previously reported that injection of glutamate (Glu) into the masseter muscle of healthy subjects causes experimental muscle pain with features comparable to the pain suffered in myofascial temporomandibular disorder (TMD) [1]. Specifically, intramuscular injection of Glu produces pain in healthy subjects that is not different in intensity and quality from pain reported by patients with myofascial TMD [1]. Injection of Glu into the masseter muscle also produces punctate mechanical sensitization which is similar, though of a lesser magnitude, to that reported by myofascial TMD patients. We have suggested that experimental designs based on glutamate injection into masticatory muscle can provide an appropriate model for studying myofascial TMD pain [1].

One of the reasons that intramuscular Glu injections may replicate symptoms of myofascial TMD pain in healthy subjects is that interstitial Glu concentrations have been shown to be elevated in patients with myofascial TMD [2]. One way that Glu concentrations can be elevated is by consumption of excessive amounts of monosodium glutamate (MSG) containing foods. In healthy subjects, a single oral ingestion of 150 mg/kg of MSG resulted in subjective reports of peri-cranial muscle tenderness and increased headache [3]. Oral administration of 150 mg/kg of MSG increases interstitial Glu concentration in the masseter muscle by up to 750 % over baseline concentrations for a period of about 90 min [4]. Recent work indicates that repeated daily administration of MSG orally to healthy individuals leads to more frequent reports of adverse effect, such as headache and nausea and results in a transient but significant lowering of pressure pain threshold and tolerance in the masseter muscle [5].

The aim of this randomized, double-blinded, placebo-controlled, cross-over study was to investigate whether prior oral ingestion of MSG could alter pain and mechanical sensitivity induced by intramuscular injection of Glu into the masseter muscle. We hypothesized that increased interstitial Glu concentration in the masseter muscle after oral ingestion of MSG would increase Glu-evoked pain and jaw muscle mechanical sensitization compared to placebo.

## Materials and methods

### Participants

Sixteen healthy adult (>18 years old) participants (8 women and 8 men, mean age ± SD: 24.9 ± 4.7 years old, mean bodyweight ± SD: 62.1 ± 13.7 kg) participated in this study. They were recruited by an advertisement posted at Aarhus University and through a webpage (www.forsoegsperson.dk). Exclusion criteria were: orofacial pain, any chronic illness, *e.g.* uncontrolled hypertension, allergy to MSG, asthma, diabetes mellitus, body mass index > 25. Informed consent was obtained from all participants. The study protocol was approved by the local ethics committee (approval No. 20060040 – amendment No. 2 of March 2010) and informed consent was obtained from all participants. The study was conducted in accordance with the Declaration of Helsinki.

### Study design

This study was performed as a randomized, double-blinded, cross-over trial. Randomization was performed by a research assistant. The examiner was blinded until data collection on all participants was completed.

**Fig. 1:**
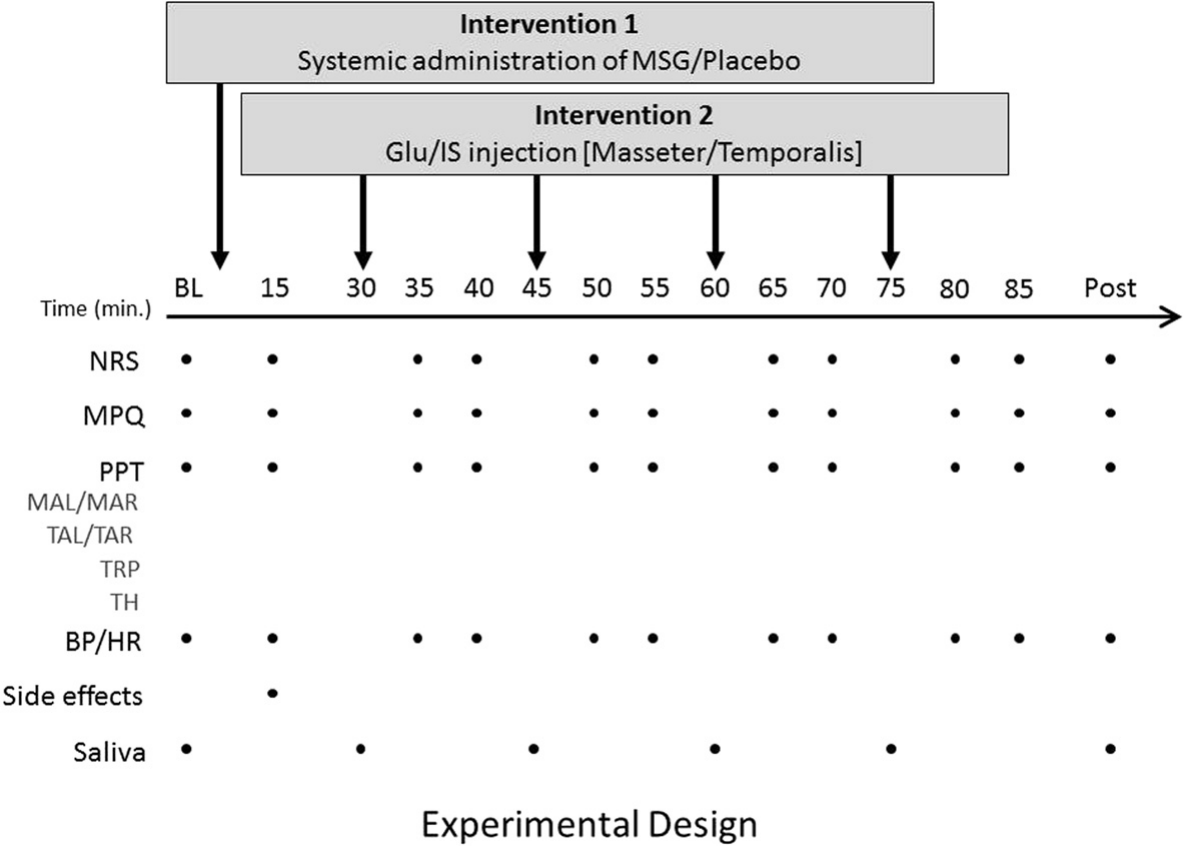
The experimental design of this study is shown. At baseline (BL), 15 min after the systemic administration, 5 and 10 min after each injection, and after again 90 min after systemic administration of monosodium glutamate (MSG) or NaCl, numeric rating scale (NRS) of pain intensity was reported by each participant. The participants were asked to draw painful area at the same time points as NRS. Pressure pain threshold (PPT) was also measured in the left and right Masseter (MAL, MAR), left and right Temporalis, the right Trapezius (TRP), the left Thenar (TH), together with systolic and diastolic blood pressure (sBP, dBP) and heart rate (HR). The participants were asked to report adverse effects 15 min after the systemic intervention. Saliva samples were collected at baseline, just before each injection was applied, and 90 min post oral ingestion of MSG or NaCl

Figure 1 shows the experimental protocol of this study. Each participant participated in 2 sessions with at least 1-week interval between sessions. The participants were asked to fast at least 2 h before the experiment [4]. Sessions began with the participants sitting comfortably on a dental chair in supine position. The participants first drank 400 mL sugar-free lemon soda (Spirit light®, Coop, Denmark) mixed with either MSG (150 mg per kg bodyweight) or sodium chloride (NaCl; 24 mg per kg bodyweight; placebo), in a randomized order [3, 4]. Experimental muscle pain in both the masseter and temporalis muscles was produced by injection of a sterile solution of glutamate (Glu: 0.5 M, 0.2 mL) 30 min after the systemic administration of MSG or placebo. On the contralateral side of the injected side in both the masseter and temporalis muscles, isotonic saline (IS: 0.9 %, 0.2 mL) was administered as a control. Four injections in total were given with 15 min interval (Fig. 1). The injections were performed manually over a 10-s period with a 27-gauge hypodermic needle and a disposable syringe [6]. Both the examiner and participants were blinded to the order and type of the injections that were given. Injection of Glu or IS were randomized for each subject. A research assistant prepared both the injections and the drink in a separate room.

### Saliva samples

To analyze the Glu concentration in whole saliva, saliva samples were collected 30, 45, 60, and 75 min after the oral administration of MSG as well as baseline and post-session. The saliva samples were collected by a commercially available collection kit, Salivette™ (Sarstedt AG & Co, Nümbrecht, Germany) [7, 8]. A kit of Salivette™ consists of a plastic tube that has a partition dividing upper and lower part, a cotton roll sheathed in the upper part of the tube, and a lid. The participants were asked to chew the cotton roll for 1 min and put it back into the tube. There was a small hole in the partition of the tube so that saliva absorbed into the cotton roll was stored in the bottom part of the tube after the tube was centrifuged at 1000 g for 2 min. The sample was stored at −80 °C. The concentration of glutamate in saliva samples was analyzed according to the previously published work [4].

### Pressure pain thresholds (PPT) and autonomic parameters

PPTs on the masseter (right and left), the temporalis (right and left), trapezius (right), and thenar (left) muscles were measured with an electronic pressure algometer (Somedic, Sweden) at baseline, 15 min after oral administration of MSG, 5 and 10 min after each injection, and at the end of the session (90 min post oral ingestion of MSG). Sites assessed for PPT on each muscle were as follows: masseter muscle; the most prominent point during contraction, approximately 2 cm superior of the mandibular border, temporalis muscle; the most prominent point during contraction in the anterior area of the muscle, trapezius muscle; the point halfway between C7 and acromion, thenar muscle; the middle of thenar eminence. The diameter of the algometer was 1 cm and rate of increase pressure was 30 kPa/s [9, 10]. The PPT was measured in triplicates at each time point. At the same time point as the PPT measurements, heart rate (HR) as well as systolic and diastolic blood pressure (sBP, dBP) were measured with a digital blood pressure monitor (UA-767 puls; A&D Medical, Abington, UK) [3, 4].

### Pain characteristics and adverse effects

All participants were instructed to rate any pain on a 0–10 numeric rating scale (NRS) at several time points: 15 min after the oral MSG administration, 5 and 10 min after the local Glu injection as well as at baseline and at the end of each session (Fig. 1). On the NRS scale, 0 indicated “not painful at all” and 10 indicated “the most pain imaginable”.

The participants were asked to draw their maximum distribution of perceived pain on lateral views (from the right and left side) of the face [11]. The pain area was digitized and expressed as arbitrary units (Sigma scan Pro 4.01.003) [1, 12].

### Statistical analysis

A paired *t*-test was performed to test a significant difference in the mean NRS scores between the Glu and IS injections. For analysis of Glu concentration in the saliva samples, a two-way ANOVA was used with session (two levels: MSG and placebo) and time (six levels: Baseline, 30, 45, 60, 75 min after the systemic intervention, and the Post) as main factors. A three-way analysis of variance (ANOVA) model for repeated measures was used to analyze the PPT values from the masseter, temporalis, trapezius and thenar muscles as well as the autonomic parameters. The factors in the ANOVA were session (two levels: MSG and placebo), type of the injections (two levels: Glu and IS), time (BL, 15 min after the oral intervention, 5 min and 10 min after the injection, and the Post). When appropriate, post hoc tests were performed with Tukey Honestly Significant Difference (HSD) test with correction for multiple comparisons. The occurrence of adverse effects was compared between sessions with Fisher’s exact test. The data are presented as mean ± standard errors of the mean (SEM). P < 0.05 was considered statistically significant.

## Results

### Experimental pain

**Fig. 2:**
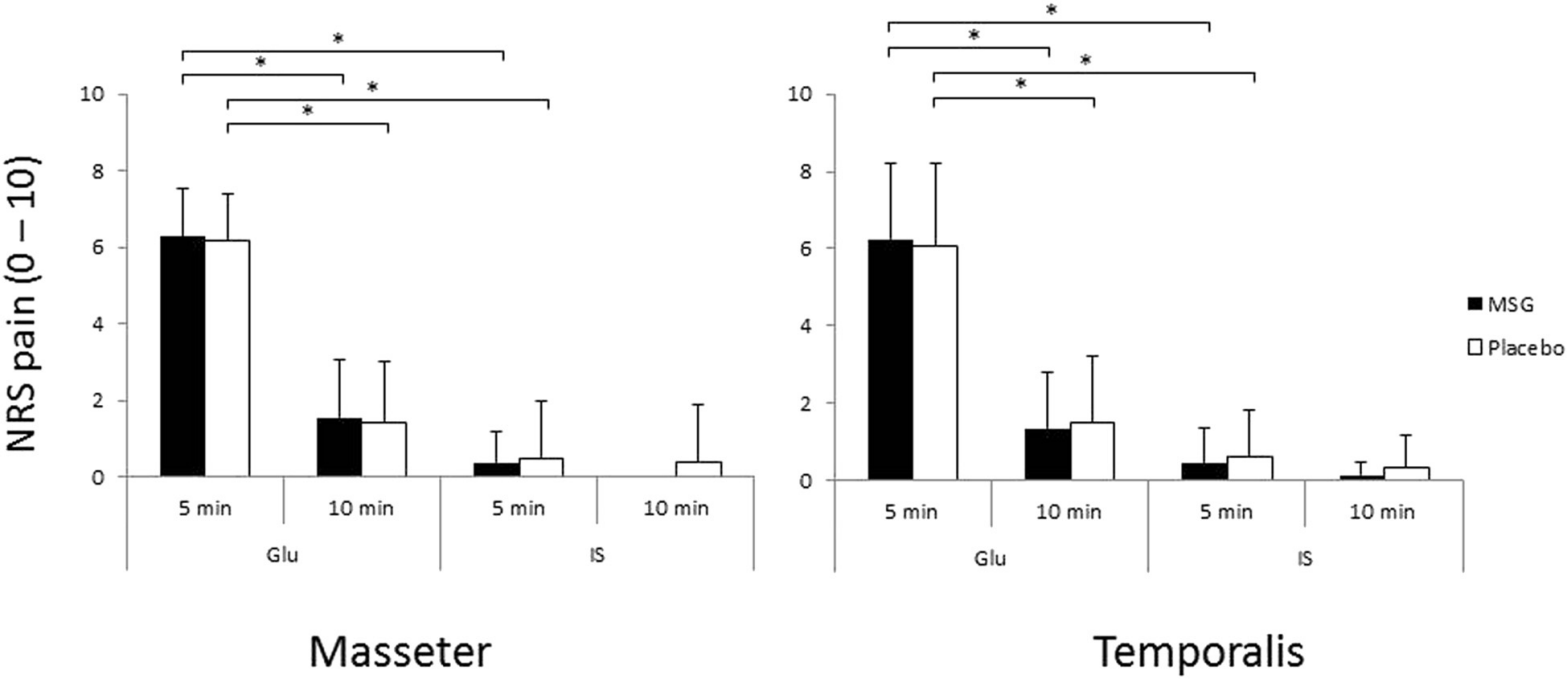
The bar graphs show the mean (±SE) pain intensity ratings 5 and 10 after injection of glutamate (Glu) or isotonic saline (IS) into the masseter or temporalis muscles. The black bars show the result from the monosodium glutamate (MSG) session, whereas the white bars show the results from the placebo session. *: P < 0.05. N = 16

**Fig. 3:**
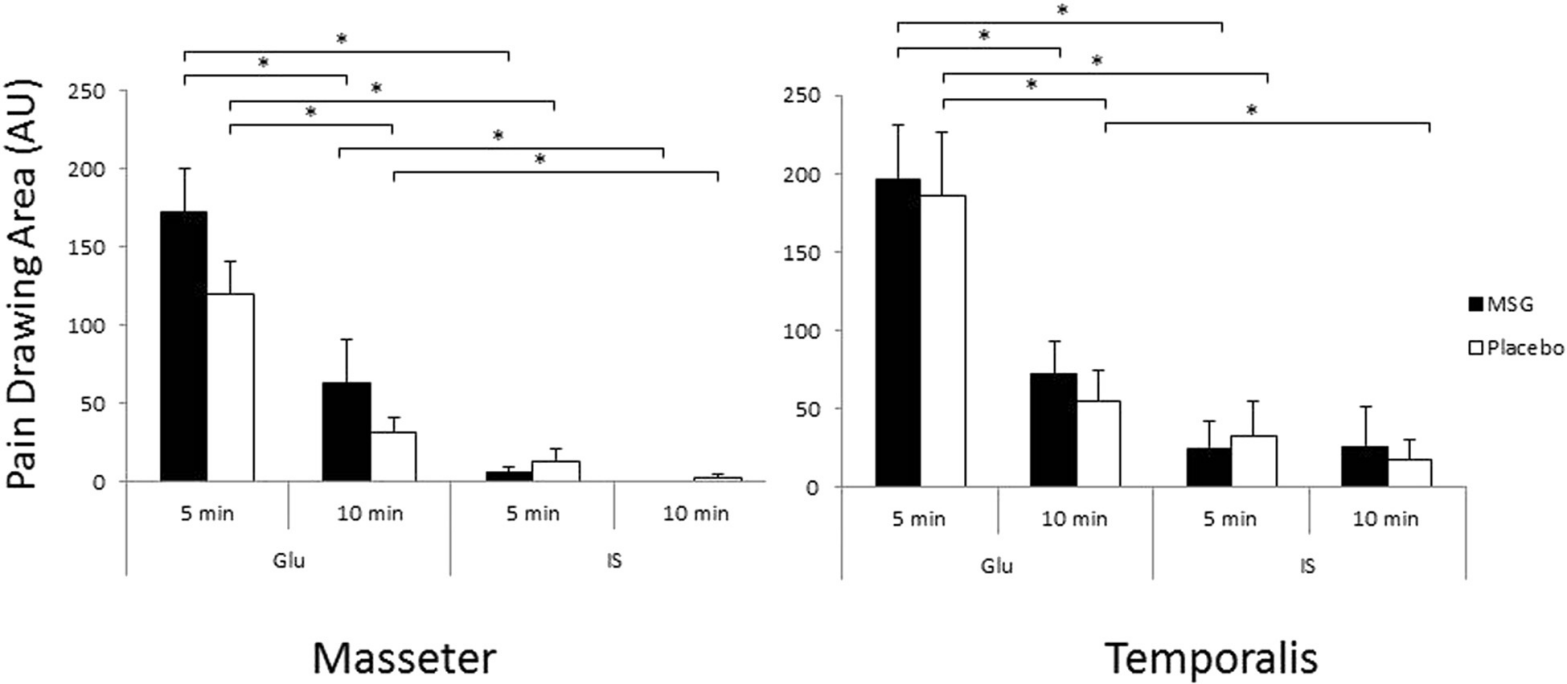
The bar graphs show the mean (±SE) areas of perceived pain drawn by the participants. Black bars shows the result of the monosodium glutamate (MSG) session, where as white bars show that of the Placebo session. *: P < 0.05. N = 16, AU = arbitrary units

In both the MSG and placebo sessions, the mean pain intensity 5 min after Glu injection into the masseter or temporalis muscles was significantly higher than the IS injection into the same muscles (P < 0.001) (Fig. 2). The pain intensity produced by Glu injections had significantly decreased by 10 min post injection in both muscles (P < 0.001). There was no significant difference in the pain intensity at any time point between the MSG and placebo sessions (P > 0.333). Glu injection into either muscle resulted in pain drawing areas that were significantly larger than those drawn after IS injection into the same contralateral muscle (P < 0.001) (Fig. 3). There was no effect of pre-ingestion of MSG on the pain drawing area (P < 0.001).

### Glutamate concentration in saliva

**Fig. 4:**
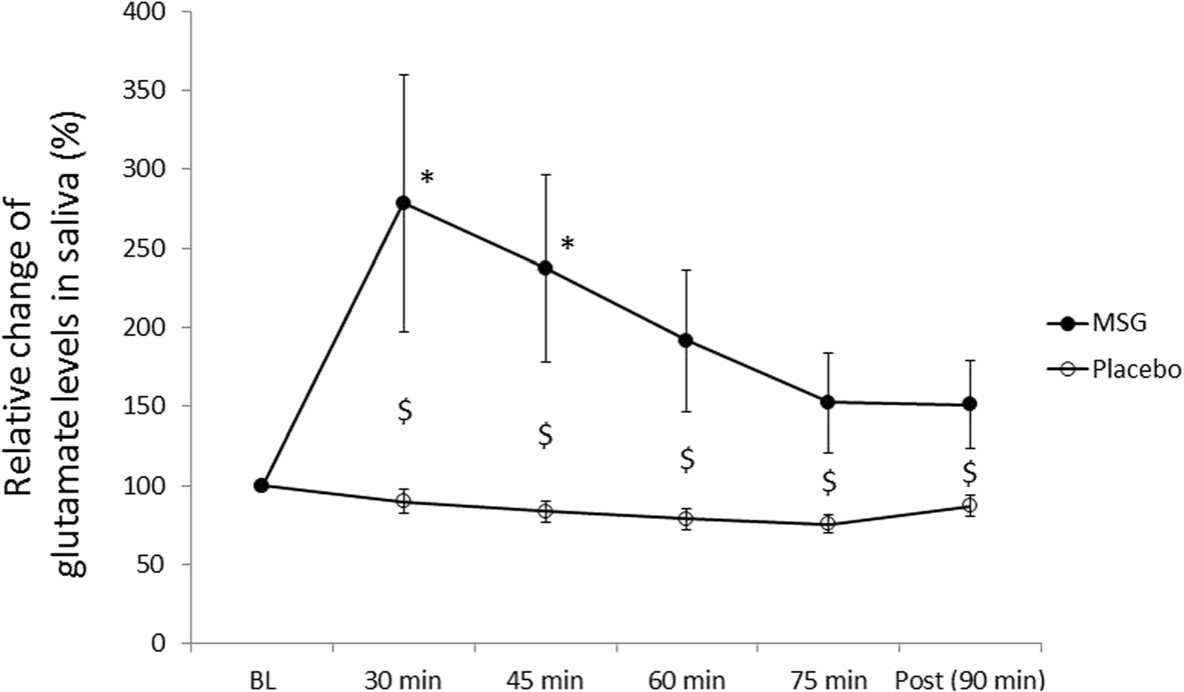
The line and scatter plot illustrates the mean (± SEM) relative changes in glutamate concentration in saliva in the monosodium glutamate (MSG) (closed circles) and placebo (open circles) sessions. P < 0.05. N = 16. * shows a significant difference compared to the baseline. $ indicates a significant difference between sessions

The ANOVA analysis of the Glu concentration in saliva samples showed a significant effect of session (P = 0.013, F = 8.656), time (P < 0.001, F = 5.406), and interaction between session and time (P = 0.004, F = 3.922). Post hoc analysis showed that the Glu concentration increased significantly 30 and 45 min after the systemic administration of MSG, compared to the baseline level (30 min: P = 0.048, 45 min: P = 0.035). Moreover, the Glu concentration in the MSG session was significantly higher than the placebo session though the entire experiment (P < 0.05) (Fig. 4).

### PPT and autonomic parameters

**Fig. 5:**
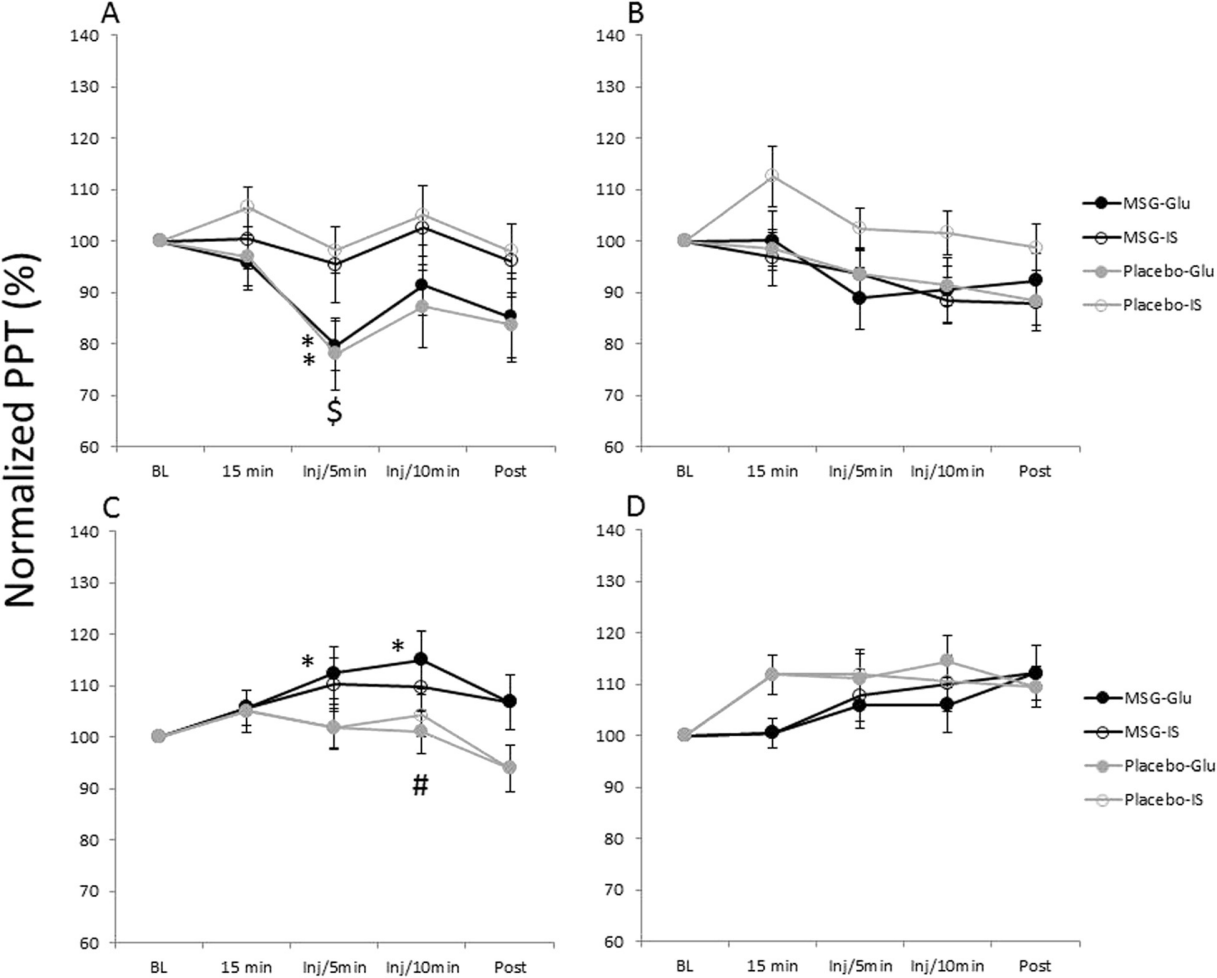
The overall mean (± SEM) relative changes in pressure pain threshold (PPT) of the masseter (Ma-ipsi: **a**) and the temporalis muscles (Ta-ipsi: **b**) on the ipsilateral side of the injection, Trapezius (**c**) and Thenar (**d**). P < 0.05. N = 16. * shows a significant difference compared to the baseline. $ shows a significant difference between the glutamate (Glu) and isotonic saline (IS) injections in the placebo session. # shows a significant difference between the monosodium glutamate (MSG) and the placebo sessions after the Glu injection

The ANOVA analyses of the PPT values and autonomic parameters (sBP, dBP and HR) are shown in Table 1. Five minutes after injection of Glu into the masseter muscle, the PPT was significantly decreased regardless of the systemic intervention (MSG: P = 0.017, Placebo: P = 0.007; Fig. 5a). In the placebo session, the PPT value in the masseter muscle 5 min after the Glu injection was significantly lower than that after the IS injection (P = 0.004). On the other hand, the PPT value in the trapezius increased significantly 5 and 10 min after the Glu injection in the MSG session (5 min: P = 0.049, 10 min: P = 0.011; Fig. 5c). Ten minutes after the Glu injection, the PPT value of the trapezius in the MSG session was significantly higher than that in the placebo session (Fig. 5c).

**Table 1:** Results of ANOVA [P value (F value)]

	Session (S)	Injection (I)	Time (T)	S x I	S x T	I x T	S x I x T
PPT							
MA	NS	0.021 (5.654)	< 0.001 (7.364)	NS	NS	0.018 (3.040)	NS
TA	NS	NS	< 0.001 (7.398)	NS	NS	NS	NS
TRP	0.045 (4.114)	NS	< 0.001 (6.839)	NS	0.001 (4.615)	NS	NS
TH	NS	NS	< 0.001 (10.012)	NS	0.006 (3.624)	NS	NS
sBP	NS	NS	< 0.001 (16.311)	NS	NS	< 0.001 (6.952)	NS
dBP	NS	NS	< 0.001 (36.462)	NS	< 0.001 (6.206)	< 0.001 (11.025)	NS
HR	< 0.001 (18.822)	NS	0.003 (4.116)	NS	< 0.001 (5.582)	NS	NS

Abbreviations

PPT: pressure pain threshold

TA: temporalis muscle

MA: masseter muscle

TRP: trapezius muscle

TH: thenar

sBP: systolic blood pressure

dBP: diastolic blood pressure

HR: heart rate

**Fig. 6:**
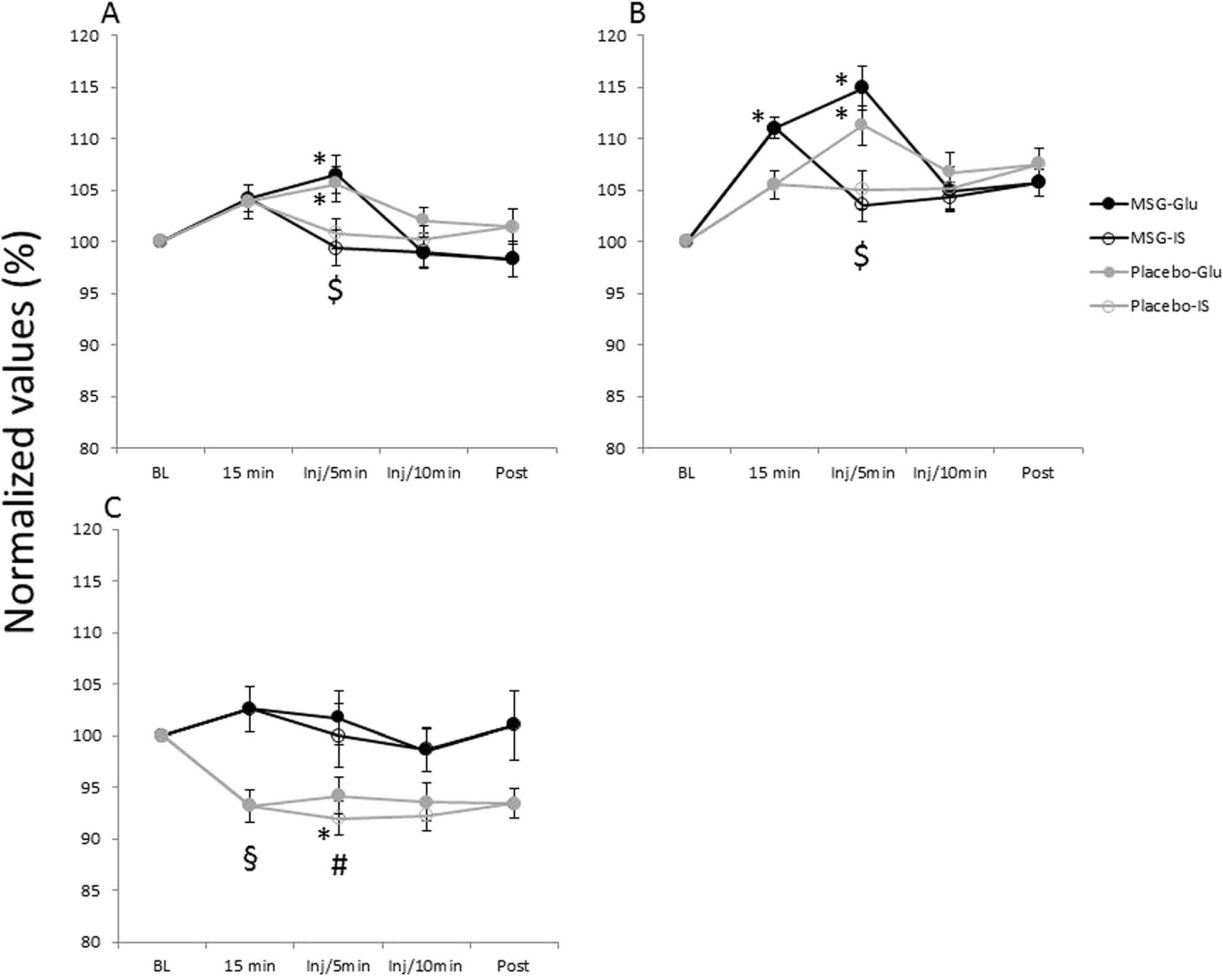
The overall mean (± SEM) relative changes in systolic blood pressure (sBP) (**a**), diastolic blood pressure (dBP) (**b**) and heart rate (HR) (**c**). P < 0.05. N = 16. * shows a significant difference compared to the baseline. $ shows a significant difference between the glutamate (Glu) and isotonic saline (IS) injections within a session. § shows a significant difference between the monosodium glutamate (MSG) and the placebo sessions. # shows a significant difference between sessions after the IS injection

The Glu injection significantly increased sBP 5 min after the injection in both the MSG and placebo session (MSG: P < 0.001, Placebo: P = 0.006; Fig. 6a). In the MSG session, sBP 5 min after the Glu injection was significantly higher than that after the IS injection (P = 0.003; Fig. 6b). There was a significant increase in dBP 15 min after the systemic MSG intervention prior to injection of Glu (P = 0.001), which lasted until 5 min after the first Glu injection (P < 0.001; Fig. 6b). dBP was also significantly increased 5 min after the Glu injection after the systemic placebo intervention, (P < 0.001). The increase of dBP 5 min after the Glu injection showed a significant difference, compared to that after the IS injection in both MSG and placebo sessions (MSG: P < 0.001, Placebo: P = 0.025; Fig. 6b). A significant effect of the systemic MSG intervention was also observed in HR (Fig. 6c). Even before the injections, 15 min after the systemic intervention, HR in the MSG session was higher than in the placebo session (P = 0.008). In the placebo session, HR 5 min after the IS injection decreased significantly, compared to the baseline (P = 0.038), which also differed significantly from that in the MSG session (P = 0.039).

### Adverse effects

The percentage of participants reporting adverse effects 15 min after systemic administration are shown in Table 2. The frequency of participants who reported dizziness in the MSG session was significantly higher than in the placebo session (P < 0.043).

**Table 2:** Occurrence of adverse effects in 16 participants (%)

	Nausea	Stomachache	Dizziness	Chest pressure	Headache	Burning sensation	Soreness in jaw muscles	Fatigue
MSG	43.8	31.3	31.3	25.0	12.5	12.5	6.3	6.3
Placebo	12.5	6.3	0.0	0.0	0.0	0.0	0.0	0.0
	NS	NS	P < 0.043	NS	NS	NS	NS	NS

## Discussion

The present study was performed to investigate whether MSG consumption alters muscle pain and mechanical sensitivity produced by an experimental pain stimulus that has some of the characteristics of muscle pain reported by myofascial TMD patients. The main finding in this study was that systemic intake of a substantial amount of MSG does not influence either pain intensity or pressure pain sensitivity in the masseter and temporalis muscles into which Glu injections were made. Unexpectedly, the systemic intake of MSG decreased mechanical pain sensitivity in the trapezius muscle after Glu was injected into either the masseter or temporalis muscles.

### Methodological considerations

In a previous study, healthy subjects who ingested 150 mg/kg of MSG had significantly elevated interstitial concentrations of Glu in their masseter muscles between 40 and 80 min after ingestion [4]. The average peak masseter muscle interstitial concentrations of Glu after a single ingestion of MSG was ~ 75 μM, and the Glu concentration remained above 50 μM for at least 60 min [4]. The present study was designed so that all four intramuscular injections were applied while interstitial Glu concentrations in the masseter would be significantly higher than baseline. Although there was no correlation between peak levels of Glu in dialysate samples and saliva samples, Glu concentrations in saliva and muscle both increased after oral administration of MSG in the previous study [4]. Therefore, the salivary Glu levels measured in this study could differentiate the MSG and placebo sessions, and indicate that subjects would have had substantially increased muscle interstitial concentrations of Glu after ingestion of MSG. Thus, the lack of difference in pain intensity and mechanical sensitization when the two sessions were compared is unlikely to be a result of failure of the subjects to be exposed to increased systemic levels of Glu. Further, it has been shown in rats that when the concentration of interstitial Glu in the masseter muscle was increased to ~75 μM, mechanical sensitization of the muscle nociceptors was maintained until the concentration dropped below 30 μM [13]. However, in previous studies in healthy subjects, a single oral dose of MSG (150 mg/kg) did not result in a significant lowering of the PPT in the masseter or temporalis muscle, but subjects did report subjective pericranial muscle tenderness [3, 5]. It is possible that in healthy human subjects, glutamate concentrations in the masseter muscle would need to be greater than those produced by ingestion of a single dose of 150 mg/kg MSG to produce measurable changes in PPT. This may have contributed to the lack of difference in PPTs in the masseter and temporalis muscles when oral consumption of MSG and placebo were compared.

It is possible that injection of a different noxious substance, rather than Glu, into the masseter or temporalis muscle might have resulted in a different outcome. For example, the pain intensity and area of pain drawings were both significantly greater when capsaicin was injected into the masseter muscle 25 min after injection of Glu (1 M), when compared with injection of isotonic saline [14]. However, no difference in mechanical sensitization was seen when capsaicin injections preceded Glu injections. There is also the potential for prolonged maintenance of elevated Glu concentrations in skeletal muscles to cause desensitization of Glu receptors after ingestion of MSG [14, 15], which might explain why we failed to see an effect of MSG on Glu evoked masseter and temporalis muscle pain. In anesthetized rats, repeated injection of Glu into the same temporomandibular joint at intervals of less than 30 min resulted in substantially decreased reflex jaw muscle responses, which suggests that clearance of Glu out of the muscle may be required to restore full nociceptive responses [16].

It needs to also be considered that only healthy subjects participated in this study. As already discussed, it is possible that glutamate concentrations in the muscles of healthy individuals were not elevated sufficiently to produce measurable mechanical sensitization after a single ingestion of MSG. Nociceptors in the masticatory muscles of TMD patients with chronic muscle pain might be far more sensitive to changes in interstitial glutamate concentrations than those in healthy subjects.

### Effect of systemic glutamate administration on perceived pain intensity and PPT

Among various risk factors for TMD, we hypothesized that daily food consumption, especially excessive MSG intake, might change muscle sensitivity to experimental pain. A significantly higher interstitial concentration of Glu [2] could trigger severe muscle pain more often in TMD patients than the healthy individuals. However, the systemic administration of MSG in this study did not result in higher glutamate-evoked pain intensity, compared to placebo. Also, consistent with past studies, a lower value of the PPT in the masseter was observed in this study, but this occurred regardless of the session [17, 18]. Thus, in this model of TMD-like myofascial pain, we found no evidence that MSG alters pain sensitivity.

The trapezius muscle is one of the most reported referred pain areas in patients with TMD [19]. Glu injection into the craniofacial region caused decreased sensitivity to pressure stimuli in the trapezius muscle after ingestion of MSG. Hypoalgesia in a heterotopic site of painful muscles has been previously observed [20, 21]. Glu evoked pain may have recruited diffuse noxious inhibitory control mechanisms (DNIC), which could have decreased the excitability of spinal dorsal horn neurons that receive sensory input from the trapezius muscle [22]. This mechanism could explain the increase in the PPT in the trapezius muscle, even though it is located outside the painful area in the temporalis or masseter muscle. However, it is also possible that this finding of hyposensitivity in the trapezius muscle could be biased by a type II error and the multiple comparisons of PPT data.

### Effect of MSG administration on autonomic parameters

In previous studies, it has been found that oral ingestion of 150 mg/kg MSG increases sBP by 5-10 % for 30–60 min, with a more variable effect on dBP and heart rate, although heart rate usually declines [3, 5]. In the present study, it was found that sBP and dBP were significantly increased by intramuscular injection of Glu into the masseter or temporalis muscle. It is possible that the increase observed in both systolic and diastolic blood pressure 5 min after Glu injections was due to the muscle pain produced by the injections. In rats, injection of NMDA, a selective Glu receptor subtype agonist, into the temporalis muscle was shown to significantly increase mean blood pressure [23]. This effect is likely due to a combination of the nociceptive input from the injection of NMDA into the muscle, coupled with a positive inotropic effect of NMDA on the heart after it is cleared from the muscle into the systemic circulation. It is uncertain whether the increased blood pressure observed in the present study after intramuscular Glu injection in healthy subjects is a result of pain alone, or is also reflects a positive inotropic effect of the injected Glu on the heart.

## Conclusion

The present study suggests that acute experimental intake of excessive MSG does not greatly affect muscle pain sensitivity in healthy subjects. Similar to results in previous studies, the most significant effect of systemic MSG administration was to increase blood pressure. As Glu injection into the masseter muscle can replicate some of the symptoms reported by patients with myofascial TMD, this study suggests that elevated dietary intake of MSG would likely not affect pain sensitivity in TMD patients. Future research on the relationship between MSG consumption and pain in TMD is required to confirm this assumption.
